# Switching of alternative electrochemical charging mechanism inside single-walled carbon nanotubes: a quartz crystal microbalance study†

**DOI:** 10.1039/d1ra04398f

**Published:** 2021-09-09

**Authors:** Ayar Al-zubaidi, Mikako Takahashi, Yosuke Ishii, Shinji Kawasaki

**Affiliations:** Department of Life Science and Applied Chemistry, Nagoya Institute of Technology Gokiso-cho, Showa-ku Nagoya 466-8555 Japan a.al-zubaidi.052@nitech.jp ishii.yosuke@nitech.ac.jp kawasaki.shinji@nitech.ac.jp

## Abstract

We probed electrochemical ion storage in single-walled carbon nanotubes (SWCNTs) of different diameters in two different organic electrolytes using electrochemical quartz crystal microbalance (EQCM) tracking. The measurements showed that charge storage probed by cyclic voltammetry did not deteriorate when steric effects seemed to hinder the accessibility of counter-ions into SWCNTs, and instead proceeded predominantly by co-ion desorption, as was shown by the decrease in the electrode mass probed by EQCM. The dominant mechanism correlated with the SWCNT diameter/ion size ratio; counter-ion adsorption dominated in the whole potential range when the diameter of SWCNTs was comparable to the size of the largest ion, whereas for larger diameters the charge increase coincided with a decrease in the electrode mass, indicating the dominance of co-ion desorption. The dominance of co-ion desorption was not observed in activated carbon, nor was it previously reported for other carbon materials, and is likely switched on because the carrier density of SWCNT increases with applied potential, and maintains the electrode capacity by co-ion desorption to overcome the steric hindrances to counter-ion adsorption.

## Abstract 2

The increase in charge carrier density in SWCNTs with applied potential overcomes steric hindrance to counter-ion adsorption by switching the dominant charge storage mechanism to co-ion desorption.

## Graphical abstract

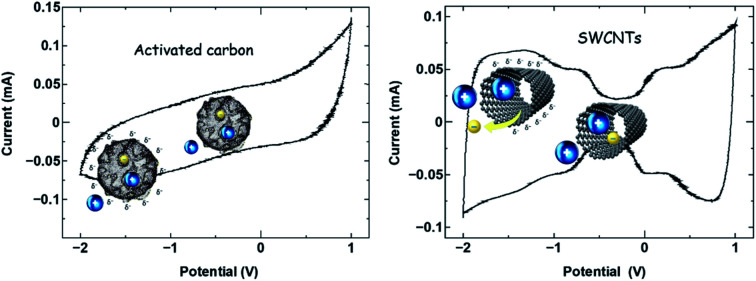

## Introduction

Electric double-layer capacitors (EDLCs) or supercapacitors have been the focus of decades of research aiming to boost their energy density to levels capable of accommodating renewable energy sources like solar and wind energy.

The quest towards high-energy density supercapacitors has relied on the development of superior electrode materials, and the deepening of understanding of the charge storage process on the electrode/electrolyte interface, either through theoretical modelling or experimental coupling of electrochemical charging with diverse *in situ* tracking techniques.^1–10^ One useful device used for *in situ* tracking is the electrochemical quartz crystal microbalance (EQCM), in which the generation of alternating current across a piezoelectric quartz crystal causes its vibration with a resonance frequency whose magnitude is sensitive to mass adhering onto the crystal. The technique has been shown to quantify the change in the mass of the electrode during electrochemical charging of an electrochemical supercapacitor,^11^ which allowed researchers to characterize the adsorption of different ionic species in porous carbon materials and obtain clues on the phenomenon of ion desolvation in pores smaller than the solvated ion size.^9,11–15^ EQCM studies have shown that the measured change in the electrode mass may be consistent with theoretically predicted values for ions with optimal accessibility into the pores, smaller to minimal under restricted conditions (smaller pores, large and/or solvated ions) suggesting the tendency for of co-ion desorption to contribute towards charge compensation (the so-called ion exchange phenomenon), or sometimes larger, which has been ascribed to the additional mass of solvent molecules accessing the pores with the adsorbed counter-ions. The degree of accordance with theoretical values did not always move monotonically but rather appeared to correlate with the magnitude of applied potential and depend on the relation between the ion and pore size. The trends were also consistent with the electrode capacitance observed by cyclic voltammetry (CV), such that minimal mass change probed by EQCM coincided with the deterioration in stored charge on the cyclic voltammogram.^11,12,16^

Unlike conventional carbon materials, single-walled carbon nanotube (SWCNTs) are characterized by a unique charge carrier density that has been shown to change with applied potential and reflect on their electrochemical charge storage behaviour.^17–32^

The cyclic voltammogram for well-crystallized SWCNTs of narrow diameter distribution is not the typical rectangle observed with other carbon materials, but resembles a dumbbell with a “grip” region that correlates with the energy gap on the density of states (DOS) of the SWCNTs, followed by a step-like increase in the capacitance when the potential passes through the van Hove singularities on each side of the energy gap.^33^ This increase results from the change in carrier density and should provide additional driving force to attract counter-ions and enhance charge storage. Previous EQCM measurements for SWCNTs in aqueous and organic electrolytes revealed a complex charge storage behavior, especially in the presence of redox activity.^15^ In particular, the adsorption of hydrated ions onto SWCNTs has been shown to depend on the pH value in aqueous solutions.^34^ Here, we expand on these observations by performing a systematic investigation through EQCM and CV measurements for SWCNTs with good crystallinity and three different diameter sizes (hence DOS profiles), in two organic redox-neutral electrolytes.

## Experimental

Three samples were used in the present study, and will be referred to as SWCNT1.0, SWCNT1.5, and SWCNT2.5 for intuitive reference to the mean SWCNT diameter in each sample. The samples went through a series of acid and heat treatments to remove amorphous carbon and metallic catalyst particles, annealed to close any defects on the walls of the tubes and improve their crystallinity, then their ends were opened by heat treatment in air.^33,35,36^ The samples were then characterized using Raman spectroscopy (Fig. S1†), X-ray diffraction (XRD), and nitrogen adsorption measurements. As explained in the ESI,† the diameter range of SWCNTs was calculated from the Raman spectra of the samples, and the mean diameters for SWCNT1.5 (1.47 nm) and SWCNT2.5 (2.48 nm) were obtained from XRD patterns assuming a triangular bundle lattice (Fig. S2†), and were consistent with the diameter range estimated from the Raman spectra. The diffraction pattern for SWCNT1.0 did not show any peaks, so we used the diameter range obtained from the Raman spectra (0.91–1.36 nm) instead. Candidate chiralities and DOS profiles for the three samples were obtained from the Kataura plot^37^ and plotted in Fig. S3–S5 of the ESI.†

Electrochemical quartz crystal microbalance (EQCM) measurements were performed in parallel with cyclic voltammetry to probe the mass change associated with the change in electrode potential. The measurements were made using two different organic electrolyte solutions, 1.0 M triethylmethylammonium tetrafluoroborate in propylene carbonate (TEMABF_4_/PC), and 1.0 M tetrabutylammonium tetrafluoroborate in propylene carbonate (TBABF_4_/PC). The details of the experimental and calculation methods are given in the ESI.†

The bundle structure of SWCNTs and the calculated mean diameter resulted in the interstitial radius values given in Table S1.† The values dismiss the possibility adsorption of ions with their solvation shells in the space between the SWCNTs, so we will focus on the hollow cores of the tubes as the main adsorption sites where the ion-to-tube size relation is likely to be of relevance to the charge storage process.

## Results and discussion

**Fig. 1:**
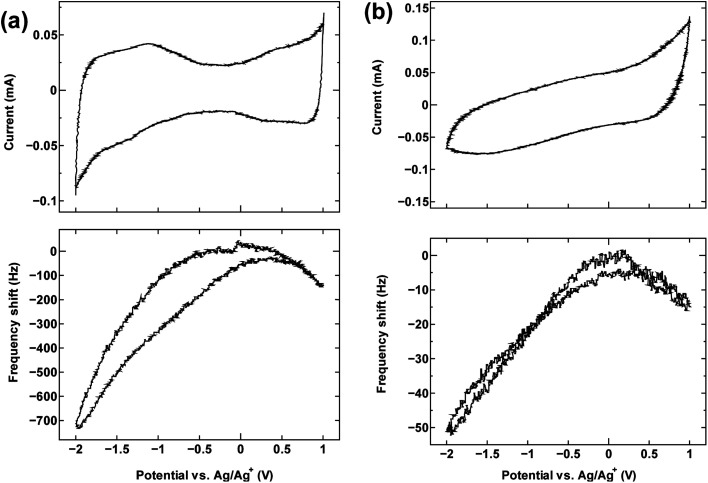
The cyclic voltammogram measured at 10 mV s^−1^ (top) and QCM frequency shift (bottom) for (a) SWCNT1.0 and (b) activated carbon in TEMABF_4_/PC.

**Fig. 2:**
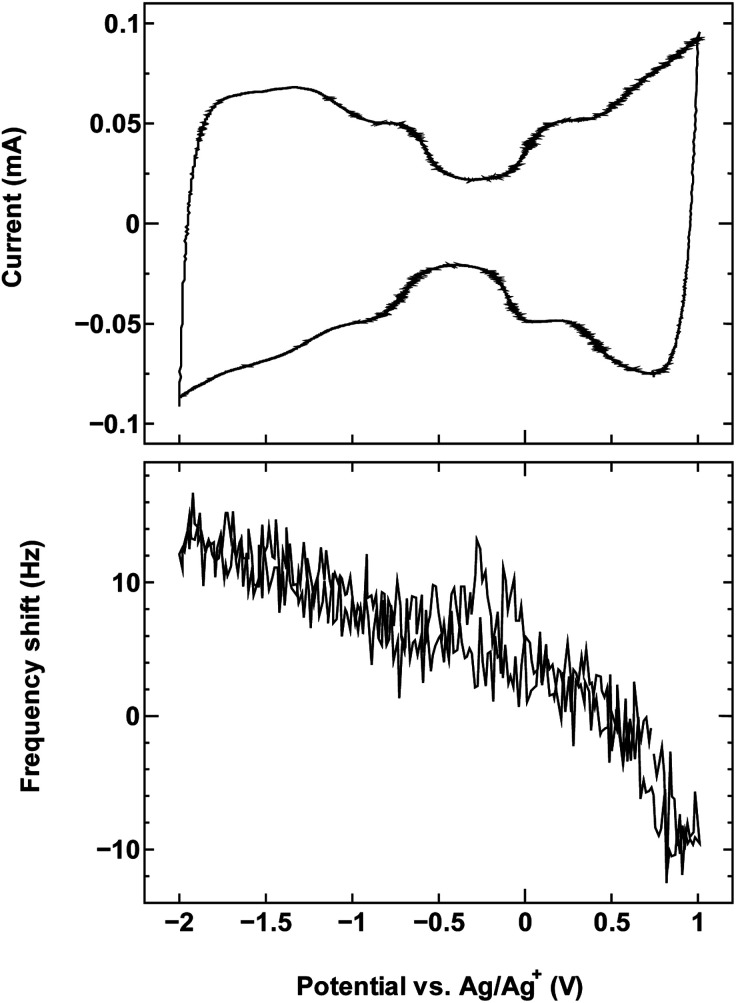
The cyclic voltammogram measured at 10 mV s^−1^ (top) and QCM frequency shift (bottom) for SWCNT1.5 in TEMABF_4_/PC.

The cyclic voltammetry curves for the three samples (top panels in Fig. 1a, 2 and 4) reveal the characteristic dumbbell shape that correlates with the electronic structure of SWCNTs. The QCM frequency shift was measured and plotted against the applied potential in the lower panel of each figure.

Fig. 1a shows the measurement result for SWCNT1.0 in TEMABF_4_/PC, along with that of activated carbon (Fig. 1b) for comparison.

The potential of zero charge (PZC) was located roughly in the middle of the “grip” of the dumbbell voltammogram (−0.13 V). The decrease in frequency (increase in electrode mass) on both sides of the center of the grip correlates with the increase in the anodic and cathodic potential on the cyclic voltammogram, and indicates that the charge storage mechanism is dominated by counter-ion adsorption in the pores of the electrode, although the contribution of co-ion desorption is also possible as implied by the slight misalignment of the potential of zero charge PZC and potential of zero mass change PZM. Judging by the calculated ion sizes (Fig. S6†), the estimated diameter size of SWCNT1.0 is expected to accommodate both the TEMA^+^ (0.84 nm) and BF_4_^−^ (0.45 nm), and allow smooth ion movement into and out of the tubes upon charging and discharging, leading to the correlation between applied potential and frequency/mass change in Fig. 1a. Activated carbon examined in the same electrolyte (Fig. 1b) showed similar qualitative frequency shift trend, but a typical rectangular cyclic voltammogram observed for 3D materials, which deteriorated at higher potentials indicating inferior current response.

The results for sample SWCNT1.5 in TEMABF_4_/PC (Fig. 2) show a different pattern, with the increase in the electrode mass (decrease in frequency) seen only in the anodic region of the voltammogram where charge storage occurs by anion adsorption, whereas the mass of the electrode in the cathodic region decreases with polarization, then recovers with depolarization.

**Fig. 3:**
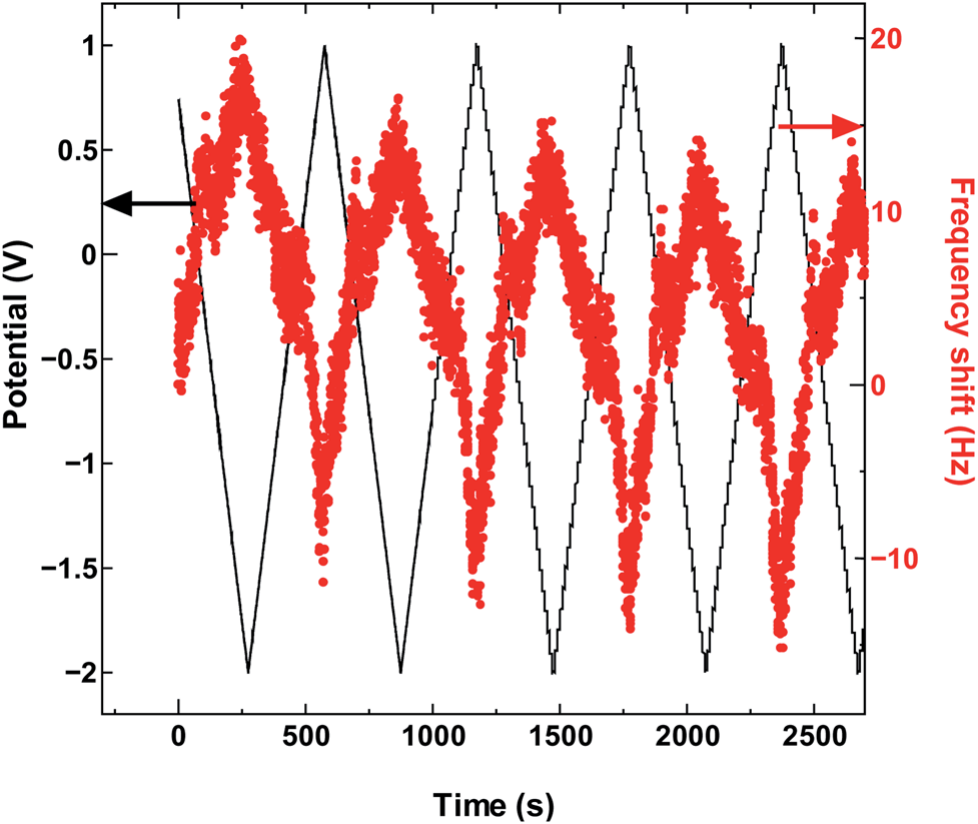
The QCM frequency shift with continued charge and discharge cycling of SWCNT1.5 in TEMABF_4_/PC.

The absence in mass increase indicates that the cation adsorption does not occur upon polarization, at least not to an extent detectable by the EQCM. Yet the capacitance on the voltammogram continues to increase, indicating charge storage *via* an alternative or additional mechanism, that is, anion desorption or migration away from the negatively charged electrode. Such co-ion predominance has not been reported previously for other porous carbons like activated carbon and carbide-derived carbon,^11,12,16^ so we hypothesize that the dominance of charge storage by co-ion desorption is unlikely to have been driven by the increase in applied potential, but rather by the additional force arising from the increase in charge carrier density of SWCNTs at potentials beyond the dumbbell grip. Considering the size of SWCNT1.5, both ions are likely to be present inside the tubes of a sufficiently wetted SWCNT1.5 sample before polarization. The increase in cathodic potential should cause additional cation migration towards and into the pores of the electrode, but the tube size in SWCNT1.5 may not allow the radial alignment of two TEMA^+^ ions. This would hinder cation adsorption needed to balance the charge of the negatively polarized electrode, and drive the migration of the much smaller BF_4_^−^ anion away from the electrode to compensate the electrode charge. This does not happen during anodic polarization, so the inside of SWCNT1.5 should allow better rearrangement for BF_4_^−^ anions to provide the space for more anions to be adsorbed upon positive polarization. This pattern of frequency change continued consistently with cycling as seen in Fig. 3.

**Fig. 4:**
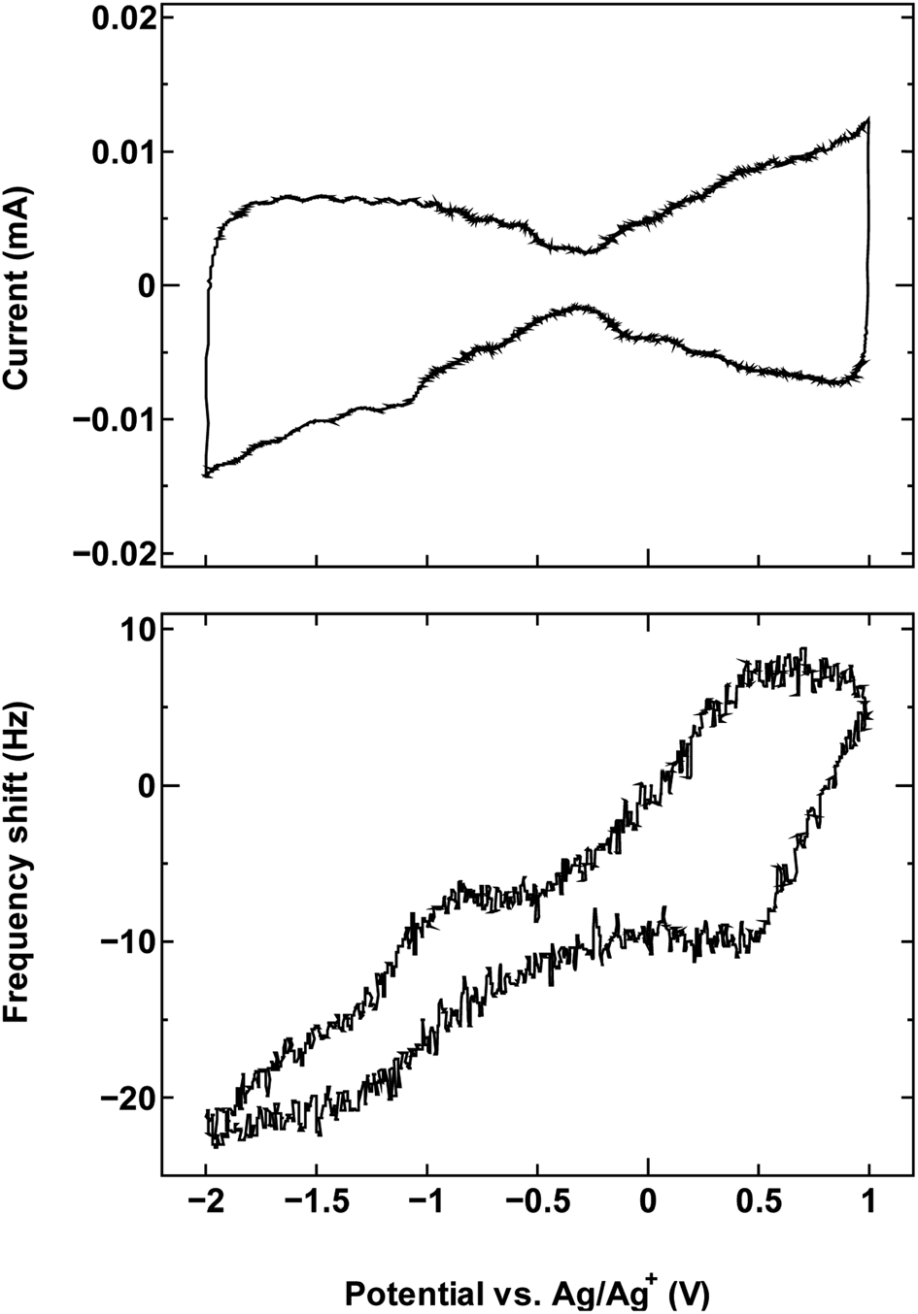
The cyclic voltammogram measured at 10 mV s^−1^ (top) and QCM frequency shift (bottom) for SWCNT2.5 in TEMABF_4_/PC.

**Fig. 5:**
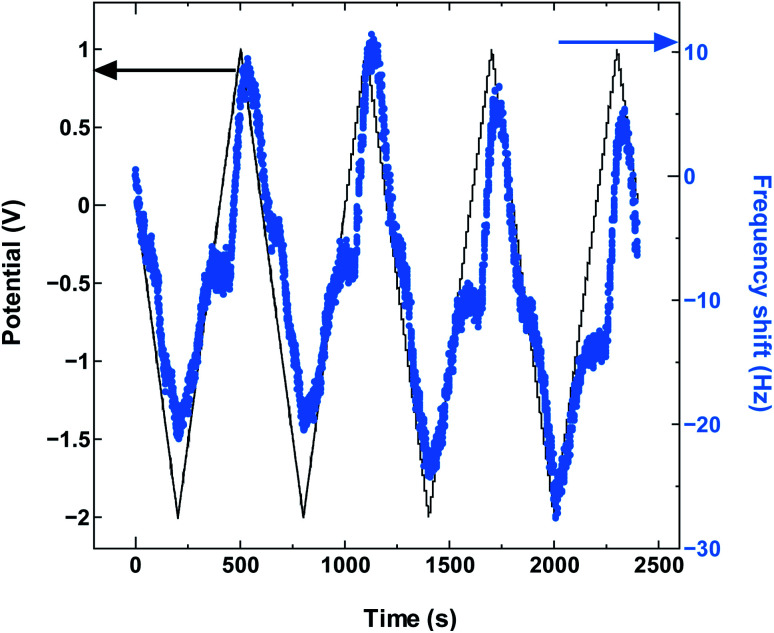
The QCM frequency shift with continued charge and discharge cycling of SWCNT2.5 in TEMABF_4_/PC.

The SWCNT2.5 sample (Fig. 4) also maintains the electrode capacitance in the whole potential range, but despite the larger tube size, the charge storage does not seem to rely solely on counter-ion adsorption in this case either. The dominance of co-ion desorption is seen again, but this time in the anodic potential region, which again is reproduced with cycling as seen in Fig. 5.

The larger average diameter size for SWCNT2.5 increases the likelihood for two TEMA^+^ cations to align themselves in the radial direction. This suggests that at PZC the cation population inside the tubes was large enough to hinder the increase in anion population in the tubes with the increase in positive potential, making cation desorption the main mechanism behind charge storage during anodic polarization.

**Fig. 6:**
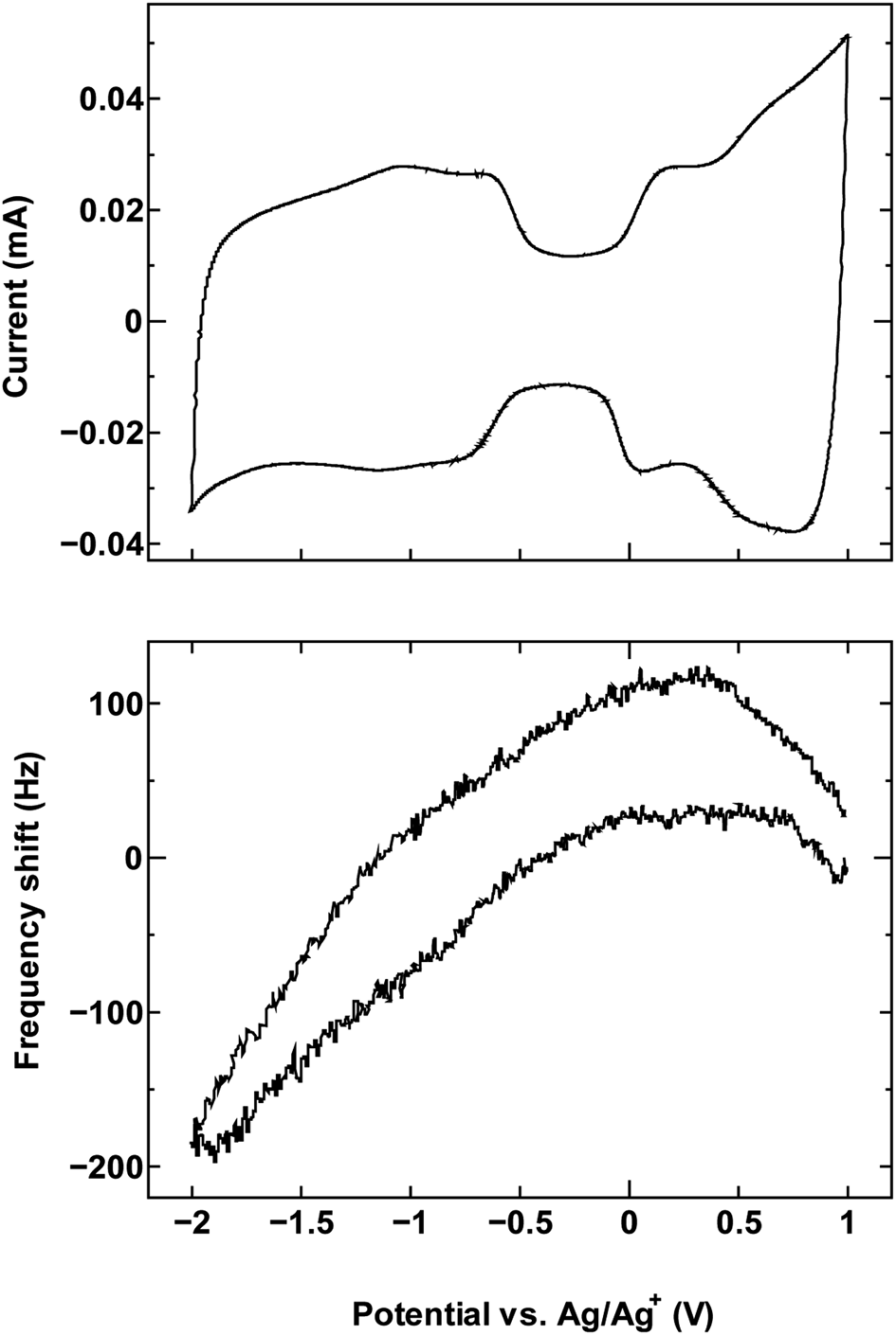
The cyclic voltammogram measured at 10 mV s^−1^ (top) and QCM frequency shift (bottom) for SWCNT1.5 in TBABF_4_/PC.

For comparison, we examined an additional SWCNT/electrolyte pairing that included SWCNT1.5 with TBABF_4_/PC (Fig. 6). The size of the cation TBA^+^ (1.1–1.2 nm) is close to the mean tube diameter in the sample SWCNT1.5, which makes the SWCNT1.5/TBABF_4_ pair comparable to SWCNT1.0/TEMABF_4_ in terms of the tube diameter/cation size ratio.

This pairing resulted in a frequency shift that is comparable to that seen in Fig. 1a, which is consistent with the ion rearrangement hypothesis and the resulting effect on counter-ion accessibility, and by consequence the dominance of a different charge storage mechanism in each case.

## Conclusions

The unique and potential-dependent carrier density in SWCNTs plays a role in maintaining the electrode capacitance even when steric hindrance may prevent counter-ion adsorption inside the tubes, giving rise to the predominance of co-ion adsorption as the main charge storage mechanism, in a manner that has not been previously seen for other porous carbon materials. The mechanism of charge storage switches to co-ion desorption when the tube-to-ion size ratio imposes constraints against the accessibility of counter-ions into SWCNTs, as has been shown from EQCM measurements where the electrode capacitance increased while the mass of the electrode dropped with polarization. The diameter of SWCNTs in the sample will allow different modes of packing for different ions, and dictate the mechanism and role of each ion in the process of charge storage.

## Author contributions

The manuscript was written through contributions of all authors, and they have all given approval to the final version of the manuscript.

## Conflicts of interest

There are no conflicts to declare.

## Supplementary Material

RA-011-D1RA04398F-s001
