# Supplementary material for: Switching of alternative electrochemical charging mechanism inside single-walled carbon nanotubes: a quartz crystal microbalance study
Source: RSC Adv. 2021 Sep 9;11(48):30253–8. doi: 10.1039/d1ra04398f (PMC9041153; doi:10.1039/d1ra04398f)
Supplement: RA-011-D1RA04398F-s001 [file RA-011-D1RA04398F-s001.pdf]

Electronic Supplementary Material (ESI) for RSC Advances.
This journal is © The Royal Society of Chemistry 2021

Switching of alternative electrochemical charging mechanism inside single-walled carbon nanotubes: a quartz crystal microbalance study

Ayar Al-zubaidi[1], Mikako Takahashi[1], Yosuke Ishii[1]*, Shinji Kawasaki[1]**

Electronic Supplementary Information

Experimental methods

Materials and characterization

The SWCNTs samples were purified by first heating in air at 350 °C for 30 minutes to remove amorphous carbon impurities, then acid washing (10 vol.% HNO_3 for SWCNT1.5 and 10 vol.% HCl for SWCNT1.0 and SWCNT2.5) to remove the metallic catalyst particles remaining from the synthesis processes. The samples were then dispersed in deionized water using a Branson 250 Analog Sonifier ultrasonic probe, and shaped into free-standing papers by vacuum-assisted filtration. To repair any defects generated by the purification treatment and improve the crystallinity of the SWCNTs, the samples SWCNT1.5 and SWCNT2.5 were annealed at 1100 °C in vacuum for 14 hours. Then the three samples went through a decapping treatment performed by heating the samples at in air for 30 minutes to open the space inside the tubes for ion adsorption. The temperature needed for decapping the tubes in each sample was determined from thermogravimetric analysis (TGA) measurements, and found to be 270 °C for SWCNT1.0, 480 °C for SWCNT1.5, and 550 °C for SWCNT2.5.[1]

Nitrogen adsorption isotherm measurements (SHIMADZU Gemini2375 analyzer) were used to determine the Brunauer–Emmett–Teller specific surface area (S_{BET}) of the samples, and to confirm the success of the decapping treatment. The S_{BET} values obtained before and after the decapping treatment (Table S1) show the increase in the surface area upon decapping.

The increase in the surface area can be deemed to originate solely from the opening of the end caps if the acid and heat treatments do not lead to the creation of defects on the walls of SWCNTs. This can be judged from the Raman spectra of the samples before and after decapping. Raman spectroscopy was performed using a JASCO NRS-3300 spectrometer with 532 nm-wavelength Nd:YAG excitation source. The obtained spectra are shown in Fig. S1. The peak around 1600 cm^{-1} is known as the G band, which is used as an indicator on the degree of graphitization of the carbon network of the SWCNT wall. The peak around 1350 cm^{-1} is the disorder-induced D band, whose intensity increases with the increase in the defects, impurities, and domain boundaries in the graphitic network.[1] All the spectra show very low D-band intensity that either remained unchanged or improved (for SWCNT2.5), which means that the samples had high crystallinity that they maintained at the end of the treatments.

The low frequency region (the left panel) of Fig. S1 shows the peak known as the radial breathing mode (RBM), whose vibration frequency is known to correlate with the diameter of the SWCNTs being probed. We estimated the diameter range of SWCNTs in the three samples from the RBM peaks using the following equation:

$$\omega_{RBM} = {}^{A}\!/_{D} + B \quad \dots \text{(S1)}$$

Here, ω_{RBM} is the frequency of the RBM peak, A = 234 cm^{-1}, and B is the upshift in ω_{RBM} caused by tube-tube interactions, and has the value 10 cm^{-1}.[2] The peak location values were obtained from fitting the spectra with a Lorentzian function using Fityk software. The instrumental limitations of

the spectrometer used in our study prevented the extension of the measureable Raman shift below 100 cm^{-1}, and that caused the spectrum for the sample SWCNT2.5 to show only a partial peak shape in the measurable RBM region. Therefore, the diameter values obtained for that sample were estimated from the extrapolation of the partial peak shape through the fitting process. The calculated diameter ranges are given in Table S1.

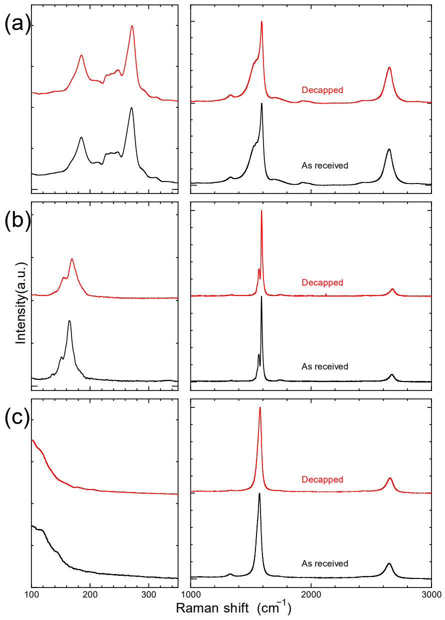

Fig. S1 Raman spectra showing the high frequency region (right) and magnified RBM region (left) for (a) SWCNT1.0 (b) SWCNT1.5(c) SWCNT2.5.

X-ray diffraction XRD experiments were performed using the beamline BL-18C at the Photon Factory (PF), High Energy Accelerator Research Organization (KEK) in Tsukuba, Japan, with incident wavelength 0.614 Å monochromatized by Si(111) double-crystal monochromator. XRD diffraction data and simulations were used to estimate the mean tube diameter and interstitial radius for each sample assuming the triangular bundle lattice shown in Fig. S2.[3] The obtained values are given in Table S1.

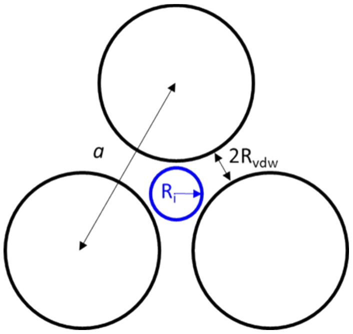

Fig. S2 The interstitial space in the triangular lattice of a SWCNTs bundle

Table S1. Properties and surface structure of the samples

Sample (product name)	SWCNT1.0 (HiPco®)	SWCNT1.5 (SO)	SWCNT2.5 (EC2.0)	AC (YP-50F)
Supplier	NanoIntegris	Meijo Nanocarbon Co., Ltd.	Meijo Nanocarbon Co., Ltd.	Kuraray Co., Ltd.
S_{BET} , annealed sample (m^2/g)	550[a]	466	970	–
S_{BET} , decapped sample (m^2/g)	730	871	1507	1293[b]
Mean diameter (nm)	0.91 - 1.36[c]	1.47	2.48	–
Bundle interstitial diameter Di (nm)	0.49 – 0.57	0.58	0.74	–
Sample weight (µg)	19.0	6.5 (in $TEMABF_4$), 14.9 (in $TBABF_4$)	10.1	89.8

a Value for the purified sample

b Value for the as-received sample

c Values calculated from RBM peak positions

As example on the unique in electronic structure of SWCNTs, we obtained candidate chiralities corresponding to the mean tube diameter of each sample, and plotted their electronic density of states in Fig. S3, Fig. S4, and Fig. S5.

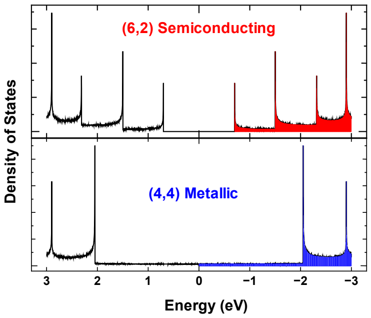

Fig. S3 The electronic density of states (DOS) for SWCNTs with diameter close to the diameter range of SWCNT1.0

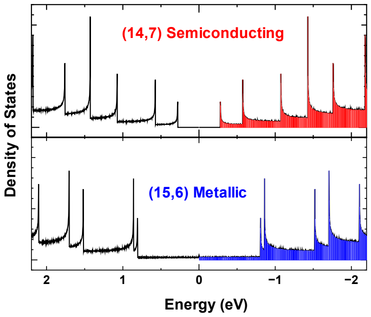

Fig. S4 The electronic density of states (DOS) for SWCNTs with diameter close to the mean diameter of SWCNT1.5

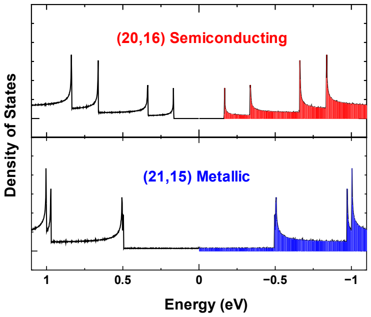

Fig. S5 The electronic density of states (DOS) for SWCNTs with diameter close to the mean diameter of SWCNT2.5

Electrochemical measurements

Two organic solutions were used as electrolytic media, 1.0 M triethylmethylammonium tetrafluoroborate in propylene carbonate ($TEMABF_4$/PC) from Toyo Gosei Co., Ltd., and 1.0 M tetrabutylammonium tetrafluoroborate in propylene carbonate ($TBABF_4$/PC) from Tokyo Chemical Industry Co., Ltd. The ion sizes for the two electrolytes were calculated from density

functional theory (DFT) calculations using Gaussian03 Software with B3LYP exchange-correlation functional and cc-pVDZ basis set. The obtained values are shown in Fig. S6.

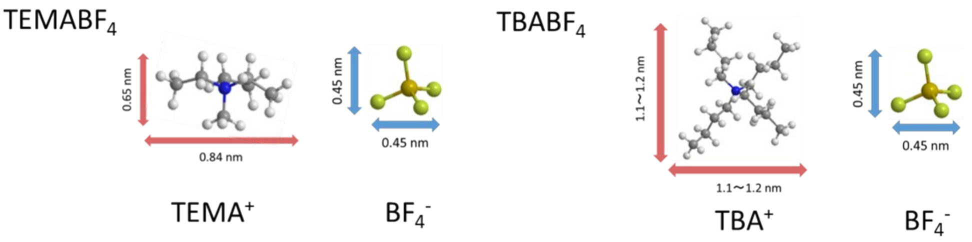

Fig. S6 The sizes of the ions of TEMABF4 and TBABF4, calculated from density functional theory

The quartz crystal microbalance system (AT-cut QCM922A microbalance from SEIKO EG&G Co., Ltd.; basic frequency 9 MHz) was used in parallel with cyclic voltammetry (SP-50CN potentiostat, Bio-Logic Science Instruments). To prepare the electrodes, the sample under evaluation was dispersed in ethanol, then drop-cast on a 5-mm diameter platinum-coated quartz substrate using a micropipette at the rate of 0.5 -1 μL. The substrate was placed on a hotplate set to 90 °C to allow the ethanol to evaporate. The weight for each sample is given in Table S1. The SWCNT-coated substrate was used as the working electrode in a three-electrode polyether ether ketone (PEEK) cell (QA-CL4PK, SEIKO EG&G Co., Ltd), with a platinum mesh as the counter electrode and Ag/Ag^+ as the reference electrode. The cell was left overnight after assembly to ensure good wetting of the electrode materials. The cell was connected to the EQCM system and the potentiostat simultaneously to measure the frequency shift during cyclic voltammetry in the potential range between -2.0 and +1.0 V at a scan rate of 10 mV/s. The measurement setup is shown in Fig. S7.

The change in the electrode mass was calculated using the Sauerbrey equation:

$$\Delta F = -\frac{2F_0^2}{A_E\sqrt{\mu\rho}}\Delta m \quad \ldots \text{(S2)}$$

In the equation above, ΔF is the change in frequency in Hz, F_0 is the initial frequency, A_E is the area of the electrode in (A=0.196 cm^2), μ is the crystal modulus of elasticity (2.947×1011 $g/cm.s^2$), and ρ is the crystal density (2.648 g/cm^3).

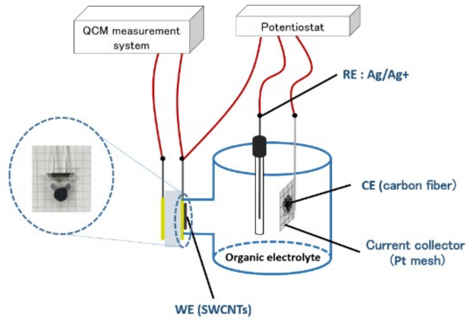

Fig. S7 Cell configuration and experimental setup for simultaneous CV and EQCM measurements

References

1 A. Al-zubaidi, N. Asai, Y. Ishii and S. Kawasaki, The effect of diameter size of single-walled carbon nanotubes on their high-temperature energy storage behaviour in ionic liquid-based electric double-layer capacitors, *RSC Adv.*, 2020, **10**, 41209–41216.

2 M. S. Dresselhaus, G. Dresselhaus, R. Saito and A. Jorio, Raman spectroscopy of carbon nanotubes, *Physics Reports*, 2005, **409**, 47–99.

3 Y. Yoshida, Y. Ishii, N. Kato, C. Li and S. Kawasaki, Low-Temperature Phase Transformation Accompanied with Charge-Transfer Reaction of Polyiodide Ions Encapsulated in Single-Walled Carbon Nanotubes, *J. Phys. Chem. C*, 2016, **120**, 20454–20461.
